# Effect of Polymer Coatings on the Permeability and Chloride Ion Penetration Resistances of Nano-Particles and Fibers-Modified Cementitious Composites

**DOI:** 10.3390/polym14163258

**Published:** 2022-08-10

**Authors:** Peng Zhang, Wenshuai Wang, Yajun Lv, Zhen Gao, Siyuan Dai

**Affiliations:** 1 Yellow River Laboratory, Zhengzhou University, Zhengzhou 450001, China; 2 School of Water Conservancy Engineering, Zhengzhou University, Zhengzhou 450001, China; 3 School of Architecture, North China University of Water Resources and Electric Power, Zhengzhou 450046, China

**Keywords:** polymer coating, cementitious composites, water contact angle, permeability, chloride ion penetration

## Abstract

Nano-particles and fibers-modified cementitious composite (NFCC) can greatly overcome the shortcomings of traditional cementitious materials, such as high brittleness and low toughness, and improve the durability of the composite, which in turn increases the service life of the structures. Additionally, the polymer coatings covering the surface of the composite can exert a good physical shielding effect on the external water, ions, and gases, so as to improve the permeability and chloride ion penetration resistance of the composite. In this study, the effect of three types of polymer coatings on the water contact angle, permeability resistance, and chloride ion penetration resistance of the NFCC with varied water–binder ratios were investigated. Three kinds of polymers (chlorinated rubber coating, polyurethane coating, and silane coating) were applied in two types of coatings, including single-layer and double-layer coatings. Three water–binder ratios of 35 wt.%, 40 wt.%, and 45 wt.% were used for the NFCC. The research results revealed that the surface of the NFCC treated with polymer coatings exhibited excellent hydrophobicity. The permeability height and chloride diffusion coefficient of the NFCC coated with different types of polymer coatings were 31–48% and 36–47% lower, respectively, than those of the NFCC without polymer coatings. The durability of the NFCC was further improved when the polymer coatings were applied to the surface in two-layer. Furthermore, it was discovered that increasing the water–binder ratio of the NFCC would lessen the positive impact of polymer coatings on the durability of NFCC.

## 1. Introduction

Cementitious composites have many advantages, such as low cost, strong plasticity, and high compressive strength, which have been favored by all kinds of constructional engineering since their inception [1]. However, a series of durability problems frequently occur in the long-term service of this material [2,3]. Due to the frequent occurrence of severe weather worldwide, the service environment for engineering structures has grown more complex and harsher in recent years. This has resulted in the premature failure of structures or components made of cementitious materials for a variety of reasons, greatly shorting the service life of engineering buildings and posing significant challenges to the durability of engineering components [4]. To improve the toughness and durability of traditional cementitious materials, the composite of the materials has become an important direction in the development of cementitious composite technology [5]. Composite methods for adding fibers or nano-particles into conventional cementitious composites have grown quickly in recent years [6].

The combination of fibers and the cementitious matrix significantly enhances the tensile strength of cementitious composites, restrains the development of cracks, and enhances the permeability resistance and toughness of cementitious composites after cracking [7,8]. Polyvinyl alcohol (PVA) fiber has high strength and elastic modulus and has the advantages of high wear resistance, acid resistance, and alkali resistance [9,10]. It has a good affinity and binding force with cement, gypsum, and other substrates and is non-toxic and pollution-free. It is an excellent and widely used artificial polymer fiber material [11]. The addition of PVA fiber can improve the properties of cementitious composites, such as crack resistance, permeability resistance, interfacial behavior, impact toughness, high-temperature resistance, and corrosion resistance [12,13,14,15]. At present, more and more nano-particles are used in the modification of cementitious composites, such as nano-SiO_2_ [16], nano-TiO_2_ [17], nano-Fe_2_O_3_ [18], nano-Al_2_O_3_ [19], nano-clay [20], etc. Among these nano-particles, the application of nano-SiO_2_ in cementitious composites is one of the earliest and most widely used nano-materials [21]. Nano-SiO_2_ has unique size effects, surface effects, and interface effects [22]. The addition of nano-SiO_2_ can enhance the internal material combination effect, effectively reduce the phenomenon of bleeding and segregation, and improve the durability and mechanical properties of cementitious composites [23,24]. Because of the complex preparation process of nano-materials and fiber materials, adding them to concrete will increase the production cost of concrete. In addition, the dispersion of fiber materials in concrete has always been a difficult problem.

The appearance of the nano-SiO_2_ and PVA fiber-modified cementitious composites is a great progress of traditional cementitious composites on the basis of modern technology and theory. It greatly improves the brittleness failure of traditional cementitious composites, enhances the durability of materials, and increases the long-term life of engineering structures [25]. However, sometimes the structures have to be served in extremely harsh and complex environments, such as freezing–thawing erosion areas, saline–alkali areas, and ocean splash areas. At this time, in order to meet the long-term reliability of engineering structures, it is necessary to further improve the durability of nano-particles and fibers-modified cementitious composite (NFCC). In addition to changing the internal structure of cementitious composites, the surface protection treatment of cementitious composites is also an effective means to enhance the durability of structures [26,27]. Polymer coatings are widely used in the surface protection of cementitious composites because of their advantages, such as economy and convenience. Polymer coatings with a wide range of applications include acrylic coatings [28], polyurethane coatings [29], epoxy coatings [30], and silane coatings [31]. In terms of the failure mechanism of the durability of cementitious composites, almost all the chemical and physical processes that affect the structural durability of cementitious composites involve the migration of water and water-borne harmful ions into the pores and cracks of cementitious composites [32]. Therefore, improving the protective performance based on surface impermeability and compactness can enhance the anti-deterioration performance of materials from the root causes of durability problems.

Polymer coatings can form a continuous polymer film with a certain thickness on the surface of the composite, and the polymer membrane can prevent the penetration of water, carbon dioxide, or chloride ion. The porosity of the coating is very low, which can effectively prevent external materials from entering the matrix through pores or cracks. In addition, the coating usually has certain air permeability, allowing water vapor to leave the cementitious matrix [33]. As a result, there is no high pressure at the interface between the cementitious matrix and coating, which leads to the cracking of the coating, ensuring the long-term effectiveness of the protective effect of the coating. Almusallam et al. [26] verified the effectiveness of the surface coating in improving the durability of cementitious composites through the durability parameters such as water absorption, chloride ion penetration, and chloride ion diffusion coefficient of cementitious composites. At the same time, it was discovered that the protective performance of epoxy coating and polyurethane coating was better than the other coatings. Sadati et al. [34] verified the long-term effectiveness of the coating through the monitoring results of chloride ion diffusion and surface chloride accumulation of cementitious composites exposed to the natural environment for 88 months. In a word, the study of the effect of polymer coatings on the durability of the NFCC is of great practical significance for their applications in cementitious composites, especially in engineering structures in harsh environments. Among the existing related studies, most of them focus on the coating protection of traditional cementitious composites; however, there is a lack of research results on the surface protection of the NFCC. Therefore, this part of the work needs to be further concerned by researchers. In this study, the surface protection treatment of cementitious composites modified by nano-SiO_2_ and PVA fiber was carried out, and the effect of different surface protection measures on the durability of the NFCC was studied by the water contact angle, permeability resistance, and chloride penetration resistance tests. The results obtained in this study can provide useful guidance for the application of the NFCC with polymer coatings in an erosive environment.

## 2. Materials and Work Method

### 2.1. Materials

**Figure 1:**
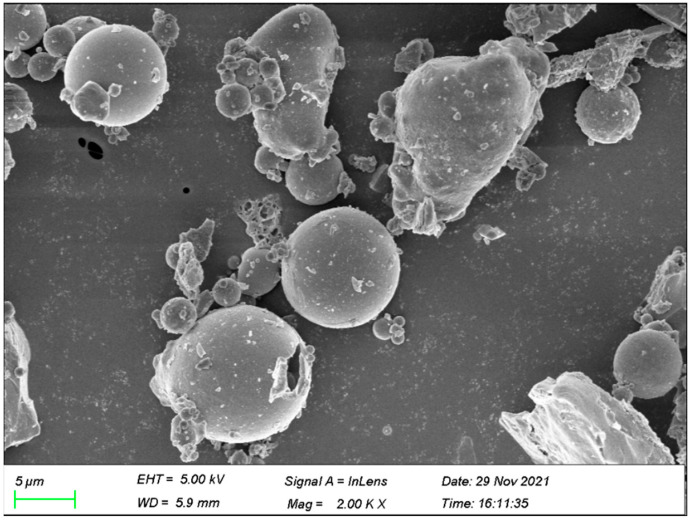
Morphology of fly ash.

Ordinary Portland cement (P·O 42.5 by GB175-2007) [35], Classifly ash [36], silica sand, water, nano-SiO_2_, PVA fiber, and a high-efficiency water-reducing agent were used to fabricate the NFCC. The fly ash used in this test was manufactured by Datang Luoyang Thermoelectric Co., Ltd. (Luoyang, China). The morphology of fly ash is illustrated in Figure 1. Table 1 lists the chemical compositions of the fly ash [37] and cement used in this study. The physical parameters of the PVA fiber and nano-SiO_2_ applied in the experiment are listed in Table 2 [38] and Table 3 [39]. The nano-SiO_2_ used in this test was manufactured by Hangzhou Wanjing New Material Co., Ltd. (Hangzhou, China). In this test, Xingchen Co., Ltd.’s (Zhejiang, China) polycarboxylic acid superplasticizer was used to ensure that the fresh NFCC had good fluidity. The silica sand had a particle size range of 212–380 μm, and the mesh size was 40–70 mesh [40]. The polymers of polyurethane, chlorinated rubber, and silane were used as the surface coating materials in this investigation. The coating polymers of chlorinated rubber and polyurethane were manufactured by Nanjing Lishui Tianlong Chemical Co., Ltd. (Nanjing, China). The commercial name for polymer coating of chlorinated rubber is J52-12 chlorinated rubber anticorrosive paint. It has a fineness of 20.8 μm and a solid content of 58% [41]. The topcoat of chlorinated rubber is a single-component coating made of chlorinated rubber resin, toughening agent, pigment, filler, and auxiliary agent, and it is red in color. The commercial name for polymer coating of polyurethane is S04-1 polyurethane anticorrosive paint. It has a tensile strength of 25 MPa and solid content of 24.2% [41]. The topcoat of polyurethane is a two-component coating consisting of low-molecular-weight carbamate polymer and a hydroxyl-containing resin, and it is blue in color. The coating polymer of silane used in the test was manufactured by Beijing Montai Weiye Building Materials Co., Ltd. (Beijing, China). The commercial name of polymer coating of silane is isobutyltriethoxy silane. Silane is a colorless liquid with a boiling point of 190 °C, a flash point of 62 °C, and a refractive index of 1.391. Its silane effective ingredient content is 98.02% and chloride ion content is 0.0036% [42].

### 2.2. Mix Proportions

To investigate the influence of the type and number of layers of polymer coatings on the durability of the NFCC, the water–binder ratios, cement–sand ratios, and the amount of nano-SiO_2_ and PVA fiber added were maintained constant. The ideal mixing proportions were obtained by varying the water–binder ratio. Two cement–sand ratios were used in this study. Fly ash and nano-SiO_2_ were added by replacing the same content of cement in mass, and the replacement rates were 35% and 2%, respectively. The PVA fiber was added to the mixture at a volume dosage of 0.9%. The amount of water-reducing agent was determined to keep the slump of each group to be consistent. The mixing proportions employed in this study are presented in Table 4.

### 2.3. Treatment of the Polymer Coating on the Specimen Surface

**Figure 2:**
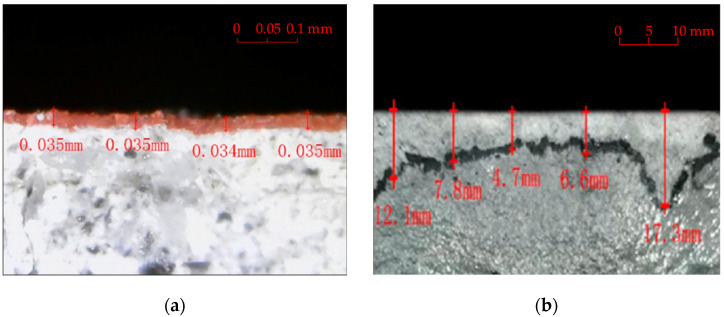
Polymer coating thickness. (**a**) Coating thickness of the chlorinated; (**b**) Coating thickness of silane rubber and polyurethane coatings.

After curing for 28 days at 20 ± 2 °C and 95% relative humidity, the specimens were treated with surface protection. Three surface-treating materials with good environmental suitability were selected for this study. The types and number of layers of polymer coatings used for the specimens prepared with three different mixing ratios are listed in Table 5. The chlorinated rubber and polyurethane coatings were coated using a wire rod coater, and the theoretical thickness of the single-layer coat was 35 μm. The amount of single-layer chlorinated rubber, polyurethane, and silane coated by a fine brush were 0.25 L/m^2^, 0.3 L/m^2^, and 0.16 L/m^2^, respectively. The cost of chlorinated rubber, polyurethane, and silane per liter is 20 CNY, 22 CNY, and 30 CNY, respectively. After the specimen was coated twice, the coating direction was vertical, and the time interval between the two layer-coatings was 2 h. The cross-sections of the polymer-coated specimens were photographed and measured using a USB microscope. As illustrated in Figure 2, the average coating thickness obtained via a single application of chlorinated rubber and polyurethane was 0.035 mm, while the average coating thickness of single-layer silane was 9.75 mm.

### 2.4. Water Contact Angle Test

**Figure 3:**
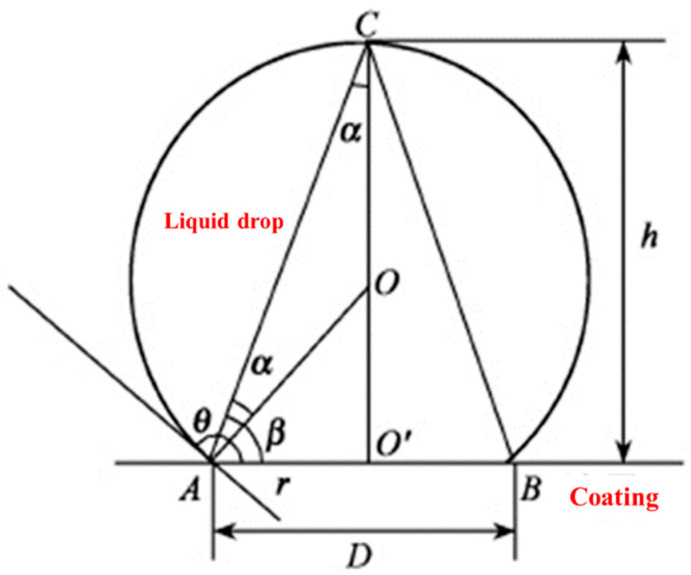
Principle of calculating water contact angle by measuring height method.

The contact angle of a liquid on the surface of solid material is an important parameter for measuring the wettability of the liquid on the material’s surface, which can be used to characterize the surface performance of the material. The effect of different polymer coatings on the hydrophobicity of the NFCC can be obtained by comparing the water contact angle values of different polymer coatings. There are many ways to measure the water contact angle. Goniometry is one of the most commonly used ones. When using goniometry to measure the water contact angle, firstly, the shape of the stable equilibrium droplet on the solid surface is observed by various instruments and equipment, and then the angle of the contact angle can be measured directly. However, goniometry is more intuitive compared to the other ways. This way will require the surveyor himself to participate in the measurement process and artificially make the tangent of the gas-liquid interface, which is difficult to ensure the accuracy of the measurement. Therefore, in order to avoid the test error caused by human factors as far as possible, the water contact angle measurement method used in this study is hypsometry [43]. Hypsometry is more accurate and faster than goniometry, and its error is smaller than that of goniometry. Although hypsometry is more accurate than goniometry, hypsometry also has errors. There are errors in the measurement of the value of H. The error of the H value comes from the selection bias of point C in Figure 3. There are also errors in the measurement of the value of D. The error of the D value comes from the selection deviation of A and B in Figure 3. Therefore, as long as we reduce the deviation of the selection of the three points in Figure 3, the accuracy of the hypsometry will be satisfactory. When the volume of the droplet is less than 6 μL, the influence of gravity on the shape of the droplet can be ignored, and the shape of the droplet can be directly regarded as a part of the standard circle. Then the water contact angle can be calculated through the shape picture. The principle of calculating the water contact angle by hypsometry is shown in Figure 3. As long as the height h of the droplet from the surface and the circumference chord D of the contact surface with the coating are measured, the contact angle θ can be calculated by Equation (1).

(1) $$\theta =2\arctan\frac{2h}{D}$$

where, θ, the static water contact angle, degree; h, the height of the droplet crown, mm; D, the circumference chord at the bottom of the droplet crown, mm.

**Figure 4:**
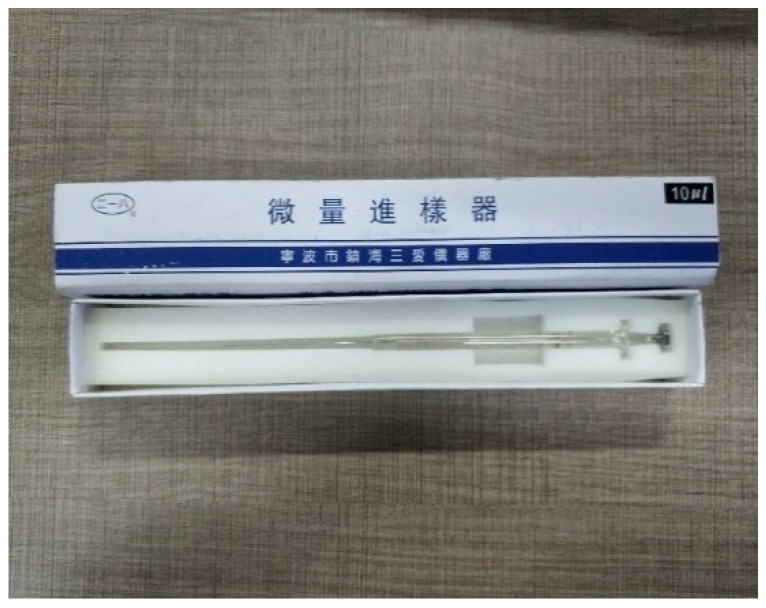
Micro sampler.

**Figure 5:**
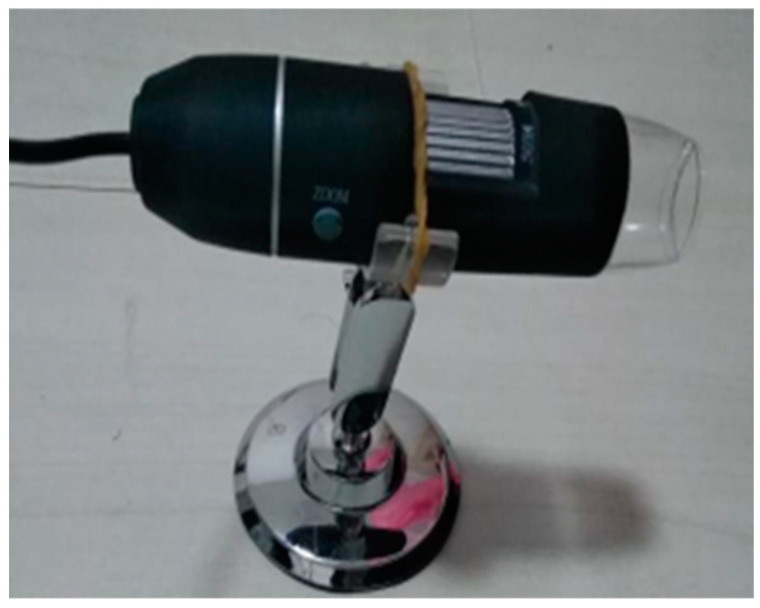
USB digital microscope.

A micro sampler (Figure 4) was used to take a drop of water drop with a volume of 5 μL, and it was dropped on the surface of the specimen with different protective treatments. A Universal Serial Bus (USB) digital microscope (Figure 5) was used to magnify the water droplets on the specimens with different surface protection by 200 times, and then the pictures were taken. According to Equation (1), the static water contact angle values of different protective coatings on specimen surface were calculated.

### 2.5. Permeability Resistance Test

**Figure 6:**
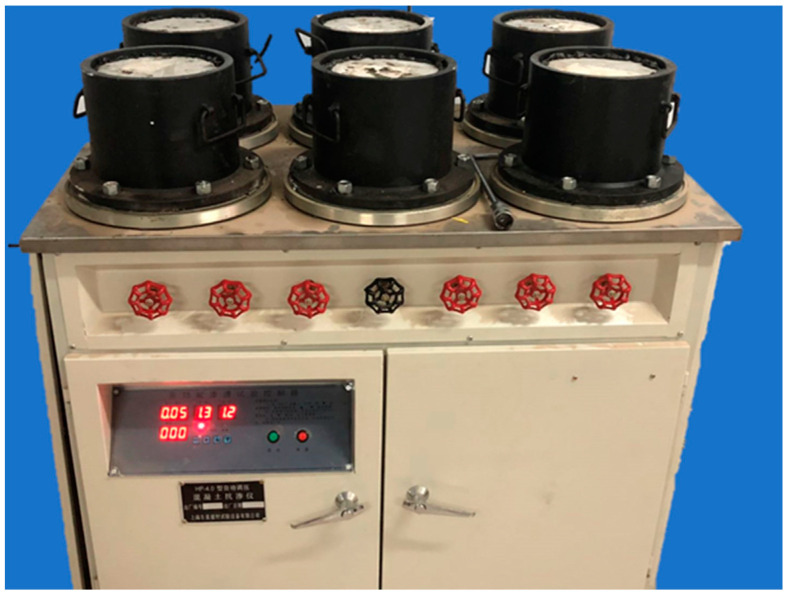
Instrument of permeability resistance of concrete.

The round table specimens with top diameter 175 mm, bottom diameter 185 mm, and height 150 mm were put into the standard curing room for curing for 28 days and then were taken out. Every six specimens were taken out as a group. The permeability surface of the specimen was treated with corresponding surface protection, and the side of the specimen was effectively sealed with paraffin wax, and then the permeability resistance test was carried out. The permeability resistance test was carried out according to the method of permeability height in GBT50082-2009 [44]. During the test, the water pressure was kept constant at 1.2 ± 0.05 MPa for 24 h, and the pressure process was controlled within five minutes. The time when the pressure value was stable was taken as the start time of the test record. The test was immediately stopped if permeability was discovered on the end face of the specimen in the process of pressure stabilization. After the permeability test, the specimen was split into two pieces in a compression testing machine. Then, the water traces were traced with a pen for waterproofing. Finally, ten permeability height values with evenly spaced measuring spots along the water marks were measured using a steel ruler. The average permeability height of the sample was determined using Equation (2). The permeability resistance test instrument is HP-4.0 type automatic pressure regulating concrete impermeability tester produced by Shanghai Dongxing Building Materials Test Equipment Co., Ltd. (Shanghai, China). The appearance of the impermeable instrument is shown in Figure 6. The maximum working pressure of the impermeable meter is 1.6 MPa.

(2) $$\bar{h_{i}}=\frac{1}{10}\sum_{j=1}^{10}h_{j}$$

where, $h_{j}$, the permeability height at the j measuring point of the specimen i, mm; $\bar{h_{i}}$, the average permeability height of specimen i, mm.

### 2.6. Chloride Ion Penetration Resistance Test

**Figure 7:**
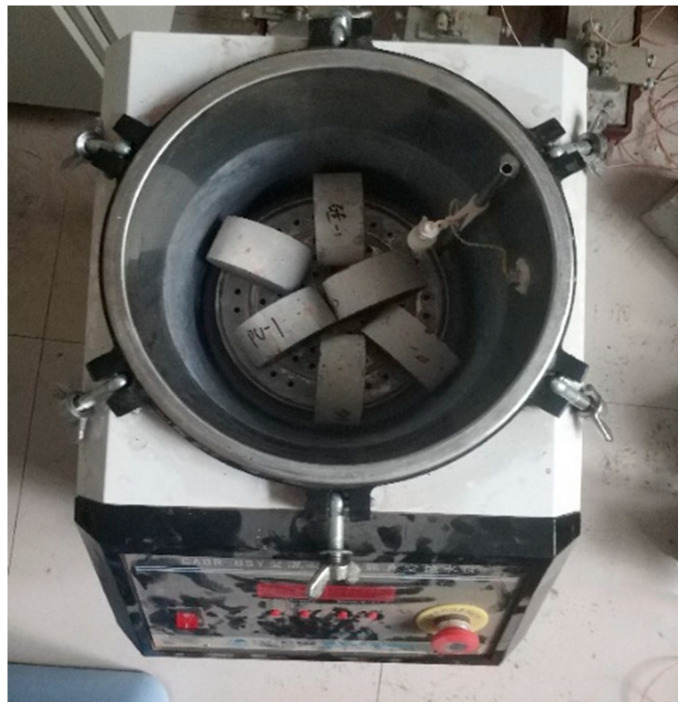
Instrument of vacuum water saturation.

**Figure 8:**
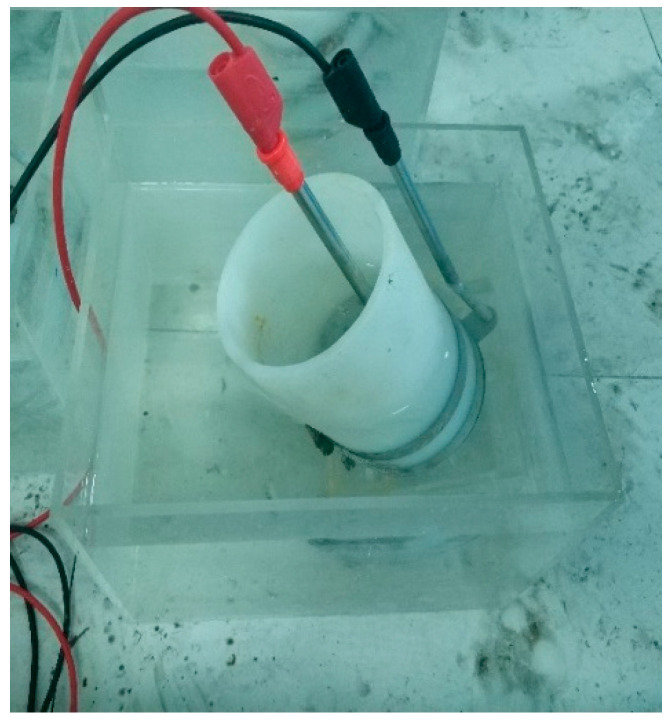
Tank of test.

**Figure 9:**
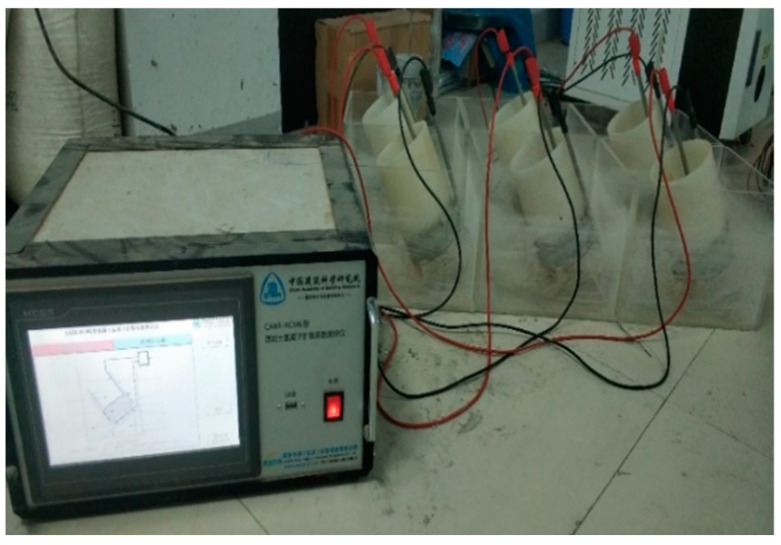
Overall device of the test.

The chloride ion penetration resistance test was carried out according to the standard steps of rapid chloride ion migration coefficient method in GB50082-2009 [44]. The chloride ion penetration resistance of the NFCC was measured by the unsteady transfer coefficient of chloride ion in the specimen. The specimen was a cylinder with a diameter of 100 mm and a height of 100 mm. The cast specimens were immersed in the pool of the standard curing room for 28 days. Before the test, a cylinder with a height of 50 mm was cut from the middle of the specimen as the test specimen, and the surface of test specimen was coated with the corresponding surface coating. At the beginning of the test, the instrument of vacuum water saturation (Figure 7) was used to vacuum water retention of the specimen. Then, the water-saturated specimen was put into the chloride ion penetration resistance test device, and the cathodic and anodic wires were connected, as shown in Figure 8. Finally, the power of the chloride ion penetration resistance tester was turned on, relevant parameters were set, and the chloride penetration resistance test was conducted. The overall test device is shown in Figure 9. The voltage was adjusted to 30 ± 0.2 V when the test was carried out, and the initial current through each specimen was recorded. The voltage was applied according to the regulations, and the initial current was recorded. The test duration was determined, and the initial temperature and final temperature of the anode solution, as well as the final current of each specimen, were recorded. After the test, the specimen was split, and the penetration depth of chloride ion was displayed by AgNO_3_ solution indicator. Then, the penetration depth was measured by digital display vernier calipers. Finally, the unsteady chloride ion diffusion coefficient was computed by Equation (3). When conducting chloride ion penetration resistance test, the determination value of the chloride ion diffusion coefficient of the specimens in each group shall be the arithmetical average value of the chloride ion diffusion coefficient of three specimens. The measured value should be the average of the other two values when the gap between the maximum or minimum value and the median value is greater than 15% of the median value. The median value should be used as the measured value when both the maximum and minimum values are greater than 15% of the median value.

(3) $$D_{RGM}=\frac{0.0239\times (273+T)L}{(U-2)t}\left(X_{d}-0.0238\sqrt{\frac{(273+T)LX_{d}}{U-2}}\right)$$

where, $D_{RGM}$, the unsteady chloride ion migration coefficient, m^2^/s; T, average temperatures of anode solution, °C; L, specimen thickness, mm; U, absolute value of voltage used, V; $X_{d}$, average penetration depth of chloride ions, mm; t, test duration, h.

## 3. Results and Discussions

### 3.1. Water Contact Angle

Surface wettability can determine whether the material surface is hydrophilic or hydrophobic. Surface wettability, which is often characterized by the contact angle formed between the droplet and the material surface, reflects the ability of the liquid dripping on the surface of a solid substance to spread out. The wettability of solid materials can be classified according to the value of the water contact angle. When the water contact angle formed by water droplets is between 10° and 90°, the material surface is called a hydrophilic surface. When the contact angle is between 90° and 150°, the surface of the material is called a hydrophobic surface. When the water contact angle exceeds 150°, the surface of the material is called a superhydrophobic surface [45].

**Figure 10:**
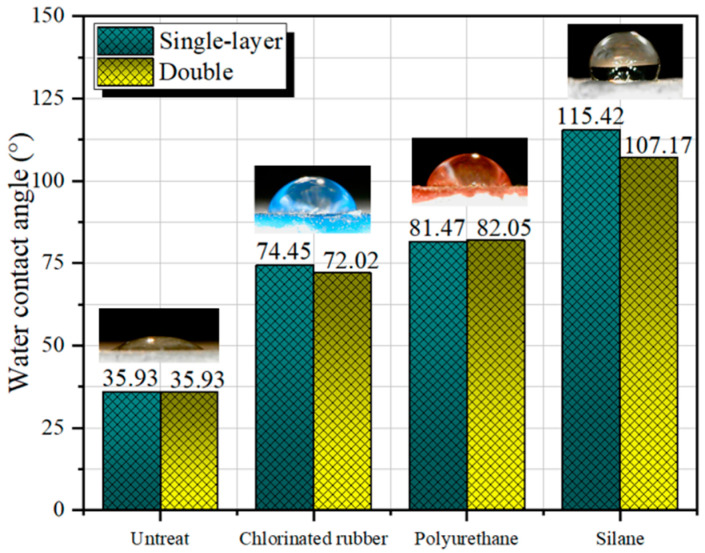
Microdroplet morphology and water contact angle of specimens (water–binder ratio 35 wt.%).

Figure 10 illustrates the morphology of trace droplets and water contact angles on the surface of specimens with water–binder ratio of 35 wt.% under the protection of various types of polymer coatings. The microdroplet height on the surface of the specimen without polymer coating protection treatment on the surface was very small, and the contact area with the specimen surface was large. On the contrary, with the treatment of polymer coating, the surface droplet shape was more rounded, which indicated that the hydrophilicity of the surface was greatly reduced, and this situation was especially obvious on the surface of the NFCC after the treatment of silane impregnation. According to the test results of the water contact angle, the hydrophilicity of the specimen surface decreased after surface protection treatment. Dong et al. [46] also proved that the polymer coating could improve the hydrophobicity of the material surface. In addition, they also found that the surface of the polymer-coated specimen remained superhydrophobic after 150 sandpaper wear cycles and 200,000 water drop impacts, which fully demonstrated the perdurability of the polymer coating. Compared with the control group without coating protection, the water contact angle of the specimen surface protected by single-layer chlorinated rubber coating and polyurethane coating increased by 107.2% and 126.7%, respectively. After impregnating the specimen with silane, the specimen surface changed from hydrophilic to hydrophobic, and the water contact angle increased by 221.2%. After adding a layer of polymer coating on the surface of the specimen, the water contact angle was tested again, and the test results showed that there was no significant difference from that of the single layer. These results indicate that the improvement of the hydrophilicity of the specimen surface by the polymer coating is related to the properties of the protective material itself, but not to the thickness of the polymer coating and the number of silane impregnation. When measuring the water contact angle of test groups with water–binder ratios of 40 wt.% and 45 wt.%, it was discovered that the results were basically consistent with those obtained from water–binder ratio of 35 wt.%, and the maximum error was no more than 3%. By analyzing this phenomenon, it can be concluded that the water contact angle of the specimen surface is mainly determined by the nature of the protective material itself and has little correlation with the specimen material itself.

The reason why surface coating protection can change the hydrophilicity of the NFCC is that the film-forming coating contains polymer resin groups, which can reduce the surface energy of the NFCC so that the surface of the material shows a certain hydrophobicity [47]. The silane and siloxane in the impregnation agent can easily penetrate into the interior of the NFCC because of their small molecular weight. The silane on the molecular chain has hydrophobic properties, which greatly reduces the surface tension of the NFCC so that the material contact angle has been greatly improved [48].

### 3.2. Permeability Resistance

Permeability resistance is one of the most important factors determining the durability of cementitious composite, which is mainly dependent on the pore structure and internal crack connectivity of the materials [49]. Because the cementitious composite is a kind of porous material [50], when the medium around the material has pressure differences, such as concentration difference, potential difference, temperature difference, etc., there will be migration of the medium subject to fluid mechanics, namely permeability. The permeability resistance not only characterizes the water resistance of cementitious composite but also affects the carbonation resistance and chloride penetration resistance of cementitious composite to a certain extent [51]. When the permeability resistance of the material is poor, it is easy to freeze and thaw at low temperatures because of water permeability. In addition, the water infiltrating into the matrix may also precipitate calcium hydroxide and other substances after a certain chemical reaction, which will result in dissolution corrosion and reducing the durability of the components. The polymer coating on the surface of the material can fill and block the pores of cementitious composites, so as to achieve the purpose of waterproofing, which plays an obvious role in improving the permeability resistance of cementitious composites.

**Figure 11:**
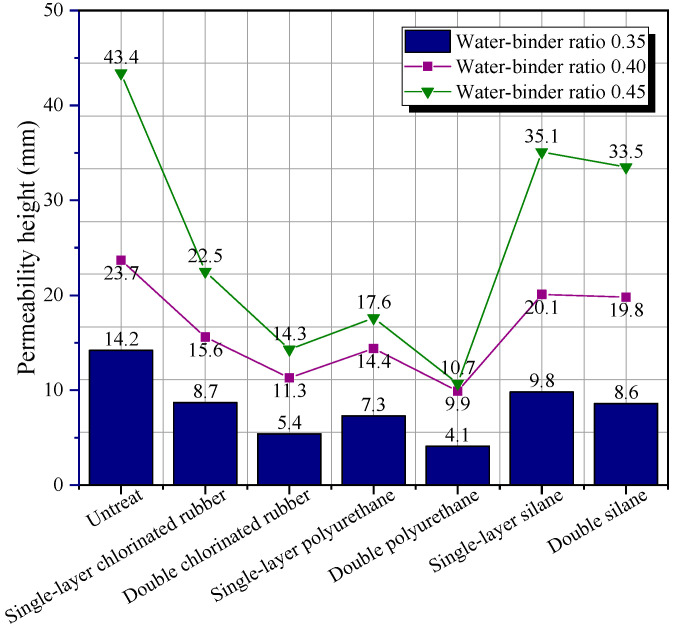
Permeability height of protective specimens with different water–binder ratios.

Figure 11 illustrates the permeability height of protective specimens with different water–binder ratios. All three kinds of polymer coatings can reduce the permeability of the cementitious composite. The improvement efficiency of polyurethane coating on the permeability resistance of specimens was the most obvious. Compared with the control group, the permeability height of the specimens with water–binder ratio of 35 wt.% decreased by 48.6% and 71.1%, respectively. The specimens with different water–binder ratios showed similar changes. In the permeability resistance test process, water penetrated into the specimen under the drive of hydrostatic pressure. After adding a layer of polymer coating on the surface of the specimen, water needed to pass through the polymer coating on the surface before penetrating into the matrix. The fact that film-forming polyurethane and chlorinated rubber coatings may produce a dense physical barrier on the surface of the material and effectively prevent water infiltration can illustrate why the three types of polymer coatings have varied effects on the impermeability of materials [52]. Polyurethane coating was more permeable resistance due to its denser structure than chlorinated rubber. However, permeable silane cannot close the pores on the surface of the specimen, so it had poor protection against water penetration.

Compared with the permeability height data of the specimens with different water–binder ratios, it was discovered that there was a negative correlation between the permeability resistance and the water–binder ratio of the cementitious composite. Diamanti et al. [33] discovered that the permeability resistance of polymer-modified cementitious materials with a water–binder ratio of 65 wt.% decreased by 50% compared with that of polymer-modified cementitious materials with water–binder ratio of 50 wt.%. This is because when the water–binder ratio increases, the compactness of the material will decrease, a large amount of free water cannot participate in the hydration reaction, and more pores and cracks will be formed after water evaporation [53]. The existence of these holes provided conditions for the infiltration of water into the matrix. Therefore, the permeability height would increase with the increase in water–binder ratio of the material. When the material surface was coated with double polymer coating, the protective efficiency of the film-forming chlorinated rubber and polyurethane coating was greatly improved compared with that of the single-layer. This is because the increase in the number of coating layers will increase the thickness of the film-forming coating, which can more effectively separate the external environment from the specimen and make the occurrence of permeability more difficult. However, increasing the number of coating layers did not change the protective mechanism of the silane coating. The silane coating still could not seal the pores and cracks on the surface of the specimen, so the protective effect was poor.

### 3.3. Chloride Ion Penetration Resistance

For cementitious composites with reinforced steel, corrosion of reinforced steel is the main cause of structural failure, and one of the primary factors contributing to the corrosion of reinforced steel is corrosion of chloride ions [54]. Chloride ions will invade the matrix of cementitious composites in various ways. When the chloride ion reaches a certain concentration on the surface of steel bars, the steel bars will be corroded by an electrochemical reaction with chloride ions. With the aggravation of corrosion of steel bar, the effective area of steel bar decreases gradually, which leads to the decrease in mechanical properties of materials [55], and when the steel bar is damaged, the volume of the steel bar will expand, leading to the cracking of cementitious composites, which will further aggravate the invasion of external chloride ions and cause more serious corrosion [56]. There are two ways for chloride ions to enter the cementitious composites. On the one hand, chloride ions can enter the cementitious composite through the usage of the admixture containing chloride ions in the preparation of the composite; on the other hand, the external chloride ions will infiltrate into the cementitious composite through cracks, pores, and other defects. For the former, it can be eliminated through strict construction management; however, for the latter, it is often difficult to eliminate the invading of chloride ions. However, the surface protection treatment on the surface of cementitious composites can block the connection between the cementitious composite and the external chloride ions to a certain extent, which can greatly slow down the penetration rate of chloride ions and improve the chloride ion penetration resistance of cementitious composites [57,58].

**Figure 12:**
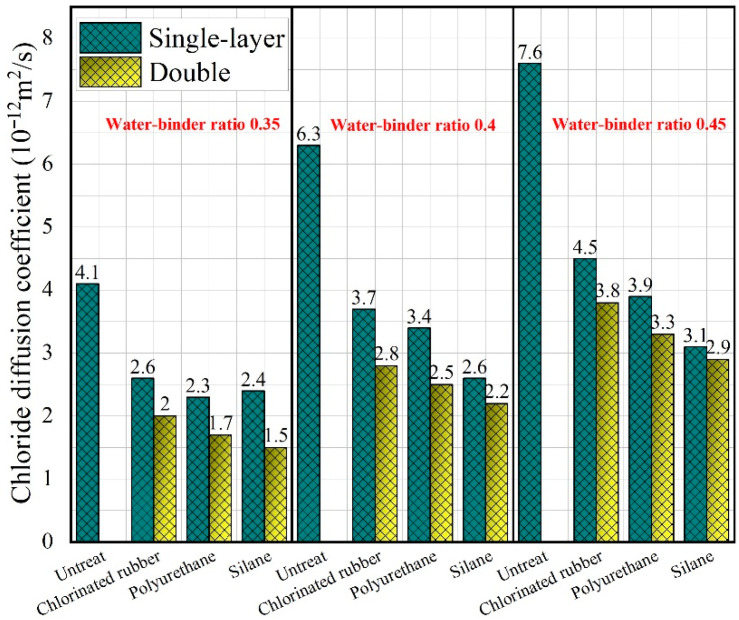
Chloride ion diffusion coefficient of the composites with different water–binder ratios coated by different layers.

The chloride ion diffusion coefficients of protective materials with varying water–binder ratios are included in Figure 12. After the protection of different types and the number of layers of polymer coatings, the unsteady chloride ion diffusion coefficient of the NFCC decreased to a certain extent. When coated with single-layer chlorinated rubber coating, polyurethane coating, and silane coating, the unsteady chloride ion diffusion coefficient of the specimen decreased by 36.6%, 43.9%, and 46.3%, respectively, compared with that of the control group. When the chlorinated rubber coating, polyurethane coating, and silane coating became two layers, the unsteady chloride ion diffusion coefficient of the NFCC was further reduced by 10–15%. Polymer coating can enhance the chloride ion penetration resistance of the NFCC. If a layer of polymer coating was added, the protective effect could be increased, and this enhancement effect was more obvious on silane coating. Compared with the application of double polymer coatings of the same type, the chloride ion penetration resistance of the specimens coated with two different types of polymer coatings will be better. Han et al. [59] found that after a layer of inorganic coating was applied again on the surface of the specimen coated with an impervious coating, and the chloride ion permeability resistance was enhanced by 37.5%. The protective effect of silane coating was superior to that of the other two coatings in both single-layer and double.

Under the protection of polymer coating, the unsteady chloride ion diffusion coefficient of the NFCC is positively correlated with the water–binder ratio of the composites. The chloride ions are mainly carried by water. Thus, if water can easily enter the interior of the material, the chloride salt dissolved in water will be more easily eroded into the material. When the water–binder ratio increases, the density of the material falls, the porosity increases, and the water is easier to penetrate into the matrix, resulting in a significant decrease in the chloride ion permeability resistance of the material [60]. Therefore, it is effective to improve the chloride ion penetration resistance of materials by adjusting the water–binder ratio reasonably and making the materials relatively denser.

## 4. Conclusions

In this study, the effect of three different polymer coatings on the water contact angle, permeability resistance, and chloride ion penetration resistance of nano-SiO_2_ and PVA fiber-modified cementitious composites was investigated. The positive influence of polymer coating on the durability of the NFCC was revealed. The main conclusions of this study were as follows:

(1) Chlorinated rubber coating, polyurethane coating, and silane coating can turn the surface of the cementitious composite into a hydrophobic surface. The surface water contact angle of the single-layer silane-coated material was 221.2% higher than that of the specimen without protective treatment. Increasing the number of coating layers and the water–binder ratio of the material had no significant effect on the water contact angle of the material surface.
(2) All three types of polymer coatings can reduce the permeability height of the composite. The polyurethane coating had the most obvious improvement in the permeability resistance of specimens. Compared with the control group, the permeability height of the specimens with a water–binder ratio of 35 wt.% coated with single-layer and double polyurethane coating decreased by 48.6% and 71.1%, respectively. There was a negative correlation between the permeability resistance and the water–binder ratios of the NFCC.
(3) The unsteady chloride ion diffusion coefficient of the NFCC decreased to a certain extent after the composites were treated with different types and numbers of layers of polymer coatings. This enhancement efficiency was more obvious for silane coating. When the number of layers of polymer coating was increased to two, the chloride ion penetration resistance of the NFCC was increased by another 10–15%.
(4) With the increase in global carbon dioxide concentration and the frequent occurrence of severe weather, carbonation failure and freeze–thaw cycle failure have become common problems threatening the durability of cementitious composites. In this paper, only the influence of polymer coating on the water contact angle, permeability resistance, and chloride ion permeability resistance of cementitious composites was studied, and the carbonation resistance and freeze–thaw cycle resistance of materials were lacking. Future research should supplement the research content on the effect of polymer coatings on carbonation resistance and freeze–thaw cycle resistance of cementitious composites.

## Figures and Tables

**Table 1:** Chemical compositions of fly ash and cement [37].

	Chemical Compositions (wt.%)							
	CaO	SiO_2_	Al_2_O_3_	Fe_2_O_3_	MgO	Na_2_O	K_2_O	SO_3_
Fly ash	9.12	52.12	17.86	6.57	3.26	2.38	2.05	0.23
Cement	63.14	21.05	5.28	2.57	3.58	0.17	0.58	2.39

**Table 2:** Physical parameters of PVA fiber [38].

Fiber Length (mm)	Fiber Diameter (μm)	Tensile Strength (MPa)	Dry Fracture Elongation (%)	Water Absorption (%)	Alkali Resistance (%)
12	40	1400	17	<1	98

**Table 3:** Physical parameters of nano-SiO_2_ [39].

Specific Surface Area (m^2^/g)	SiO_2_ Content (%)	Average Particle Size (nm)	pH Value	Bulk Density (g/cm^3^)	Heating Loss (%)	Ignition Loss (%)
200	99.5	30	6	0.055	1.0	1.0

**Table 4:** Mixing proportions of the NFCC.

Group	Water–Binder Ratio (wt.%)	Cement (kg/m^3^)	Fly Ash (kg/m^3^)	Nano-SiO_2_ (kg/m^3^)	PVA Fiber (kg/m^3^)	Silica Sand (kg/m^3^)	Water-Reducingagent (wt.%)
A	35	637	350	13	8.19	500	3
B	40	637	350	13	8.19	500	1.3
C	45	637	350	13	8.19	500	—

**Table 5:** Polymer coating types and layers used for the specimens.

Group	Type of Coating	Number of Layers
A	Chlorinated rubber coating	One Two
	Polyurethane coating	
	Silane coating	
B	Chlorinated rubber coating	One Two
	Polyurethane coating	
	Silane coating	
C	Chlorinated rubber coating	One Two
	Polyurethane coating	
	Silane coating	

## Data Availability

Not applicable.

## References

[1] Wang L., Luo R.Y., Zhang W., Jin M.M., Tang S.W. (2021). Effects of fineness and content of phosphorus slag on cement hydration, permeability, pore structure and fractal dimension of concrete. Fractals.

[2] Wu M., Johannesson B., Geiker M. (2012). A review: Self-healing in cementitious materials and engineered cementitious composite as a self-healing material. Constr. Build. Mater..

[3] Zhang P., Kang L., Zheng Y., Zhang T., Zhang B. (2022). Influence of SiO_2_ /Na_2_O molar ratio on mechanical properties and durability of metakaolin-fly ash blend alkali-activated sustainable mortar incorporating manufactured sand. J. Mater. Res. Technol. JMRT.

[4] Zhang P., Li Q.-F., Wang J., Shi Y., Ling Y. (2019). Effect of PVA fiber on durability of cementitious composite containing nano-SiO_2_. Nanotechnol. Rev..

[5] Golewski G.L., Szostak B. (2021). Strengthening the very early-age structure of cementitious composites with coal fly ash via incorporating a novel nanoadmixture based on C-S-H phase activators. Constr. Build. Mater..

[6] Murthy A.R., Ganesh P. (2019). Effect of steel fibres and nano silica on fracture properties of medium strength concrete. Adv. Concr. Constr..

[7] Abbas Y.M., Khan M.I. (2016). Fiber-matrix interactions in fiber-reinforced concrete: A review. Arab. J. Sci. Eng..

[8] Wen C., Zhang P., Wang J., Hu S. (2022). Influence of fibers on the mechanical properties and durability of ultra-high-performance concrete: A review. J. Build. Eng..

[9] Zhang P., Gao Z., Wang J., Guo J., Wang T. (2022). Influencing factors analysis and optimized prediction model for rheology and flowability of nano-SiO_2_ and PVA fiber reinforced alkali-activated composites. J. Clean Prod..

[10] Wang L., Guo F.X., Yang H.M., Wang Y., Tang S.W. (2021). Comparison of fly ash, PVA fiber, MgO and shrinkage-reducing admixture on the frost resistance of face slab concrete via pore structural and fractal analysis. Fractals.

[11] Pakravan H.R., Ozbakkaloglu T. (2019). Synthetic fibers for cementitious composites: A critical and in-depth review of recent advances. Constr. Build. Mater..

[12] Gao Z., Zhang P., Wang J., Wang K.X., Zhang T.H. (2022). Interfacial properties of geopolymer mortar and concrete substrate: Effect of polyvinyl alcohol fiber and nano-SiO_2_ contents. Constr. Build. Mater..

[13] Tan Y., Xu Z.L., Liu Z.L., Jiang J.H. (2022). Effect of silica fume and polyvinyl alcohol fiber on mechanical properties and frost resistance of concrete. Buildings.

[14] Al-Majidi M.H., Lampropoulos A.P., Cundy A.B., Tsioulou O.T., Al-Rekabi S. (2018). A novel corrosion resistant repair technique for existing reinforced concrete (RC) elements using polyvinyl alcohol fibre reinforced geopolymer concrete (PVAFRGC). Constr. Build. Mater..

[15] Gao Z., Zhang P., Guo J.J., Wang K.X. (2021). Bonding behavior of concrete matrix and alkali-activated mortar incorporating nano-SiO_2_ and polyvinyl alcohol fiber: Theoretical analysis and prediction model. Ceram. Int..

[16] Feng P., Chang H.L., Liu X., Ye S.X., Shu X., Ran Q.P. (2020). The significance of dispersion of nano-SiO_2_ on early age hydration of cement pastes. Mater. Des..

[17] Guo Z., Huang C.X., Chen Y. (2020). Experimental study on photocatalytic degradation efficiency of mixed crystal nano-TiO_2_ concrete. Nanotechnol. Rev..

[18] Nazari A., Riahi S. (2011). The effects of curing medium on the flexural strength and water permeability of cementitious composites containing Fe_2_O_3_ nanofillers. Int. J. Mater. Res..

[19] Feng H., Shen S.H., Pang Y.Y., Gao D.Y., Wang Z.Y., Sheikh M.N. (2021). Mechanical properties of fiber and nano-Al_2_O_3_ reinforced magnesium phosphate cement composite. Constr. Build. Mater..

[20] Sakr M.R., Bassuoni M.T. (2020). Effect of nano-based coatings on concrete under aggravated exposures. J. Mater. Civ. Eng..

[21] Murthy A.R., Ganesh P., Kumar S.S., Iyer N.R. (2015). Fracture energy and tension softening relation for nano-modified concrete. Struct. Eng. Mech..

[22] Han Q.Y., Zhang P., Wu J.J., Jing Y.T., Zhang D., Zhang T.H. (2022). Comprehensive review of the properties of fly ash-based geopolymer with additive of nano-SiO_2_. Nanotechnol. Rev..

[23] Singh L.P., Karade S.R., Bhattacharyya S.K., Yousuf M.M., Ahalawat S. (2013). Beneficial role of nanosilica in cement based materials—A review. Constr. Build. Mater..

[24] Szostak B., Golewski G.L. (2021). Rheology of cement pastes with siliceous fly ash and the CSH nano-admixture. Materials.

[25] Zhang X.M., Zhang P., Wang T.Y., Zheng Y., Qiu L.H., Sun S.W. (2022). Compressive strength and anti-chloride ion penetration assessment of geopolymer mortar merging PVA fiber and nano-SiO_2_ using RBF-BP composite neural network. Nanotechnol. Rev..

[26] Almusallam A.A., Khan F.M., Dulaijan S.U., Al-Amoudi O.S.B. (2003). Effectiveness of surface coatings in improving concrete durability. Cem. Concr. Compos..

[27] Muzenski S., Flores-Vivian I., Sobolev K. (2015). Durability of superhydrophobic engineered cementitious composites. Constr. Build. Mater..

[28] Easton T., Poultney S. (2007). Waterborne silicone-organic hybrid coatings for exterior applications. J. Coat. Technol. Res..

[29] Zhu Y.F., Xiong J.P., Tang Y.M., Zuo Y. (2010). EIS study on failure process of two polyurethane composite coatings. Prog. Organ. Coat..

[30] Kocijan A., Conradi M., Hocevar M. (2019). The influence of surface wettability and topography on the bioactivity of TiO_2_/epoxy coatings on AISI 316L stainless steel. Materials.

[31] Ji W.G., Hu J.M., Liu L., Zhang J.Q., Cao C.N. (2006). Water uptake of epoxy coatings modified with gamma-APS silane monomer. Prog. Organ. Coat..

[32] Barbucci A., Delucchi M., Cerisola G. (1997). Organic coatings for concrete protection: Liquid water and water vapour permeabilities. Prog. Organ. Coat..

[33] Diamanti M.V., Brenna A., Bolzoni F., Berra M., Pastore T., Ormellese M. (2013). Effect of polymer modified cementitious coatings on water and chloride permeability in concrete. Constr. Build. Mater..

[34] Sadati S., Arezoumandi M., Shekarchi M. (2015). Long-term performance of concrete surface coatings in soil exposure of marine environments. Constr. Build. Mater..

[35] (2007). Common Portland Cement.

[36] (2014). Technical Code for Application of Fly Ash Concrete.

[37] United Nation Quality Detection (2022). Chemical Compositions of Fly Ash.

[38] United Nation Quality Detection (2022). Physical Parameters of SiO_2_.

[39] United Nation Quality Detection (2022). Physical Parameters of PVA Fiber.

[40] United Nation Quality Detection (2022). Particle Size of Silica Sand.

[41] Beijing Yingjun Testing Technology Service Co., Ltd. (2022). Technical Parameters of Chlorinated Rubber Coating and Polyurethane Coating.

[42] Beijing Yingjun Testing Technology Service, Co., Ltd. (2022). Technical Parameters of Silane Coating.

[43] Wenqin D., Yingzhu W. (2007). Comparison of hypsometry and goniometry in contact angle measurement. J. Text. Res..

[44] (2009). Standard for Test Methods of Long-Term Performance and Durability of Ordinary Concrete.

[45] Hao L., Wang H., Chen R. (2019). Organic–inorganic hybrid hydrophobic Mg(OH)_2_−xFx–MTES coating with ultraviolet durability and high visible transmittance. J. Mater. Sci..

[46] Dong K., Bian L., Liu Y., Guan Z. (2022). Superhydrophobic coating based on organic/inorganic double component adhesive and functionalized nanoparticles with good durability and anti-corrosion for protection of galvanized steel. Colloids Surf. A Physicochem. Eng. Asp..

[47] Yuan T., Zhou C., Zhou J., Huang J., Tu W., Yang Z. (2016). Preparation and properties of permeable organosiloxane protective paste used for concrete protection. J. Build. Mater..

[48] Esposito Corcione C., De Simone N., Santarelli M.L., Frigione M. (2017). Protective properties and durability characteristics of experimental and commercial organic coatings for the preservation of porous stone. Prog. Organ. Coat..

[49] Li Y., Zhang J., He Y., Huang G., Li J., Niu Z., Gao B. (2022). A review on durability of basalt fiber reinforced concrete. Compos. Sci. Technol..

[50] Wang L., Lu X., Liu L.S., Xiao J., Zhang G., Guo F.X., Li L. (2022). Influence of MgO on the hydration and shrinkage behavior of low heat Portland cement-based materials via pore structural and fractal analysis. Fractal Fract..

[51] Bogas J.A., Real S. (2019). A review on the carbonation and chloride penetration resistance of structural lightweight aggregate concrete. Materials.

[52] Sun C., Kang L., Zhao X., Li W. (2016). Permeability resistance of concrete coatings. Bull. Chin. Ceram. Soc..

[53] Zhang R.L., Liu P., Ma L.N., Yang Z.J., Chen H.S., Zhu H.X., Xiao H.G., Li J. (2020). Research on the corrosion/permeability/frost resistance of concrete by experimental and microscopic mechanisms under different water-binder ratios. Int. J. Concr. Struct. Mater..

[54] Liu M., Cheng X.Q., Li X.G., Jin Z., Liu H.X. (2015). Corrosion behavior of Cr modified HRB400 steel rebar in simulated concrete pore solution. Constr. Build. Mater..

[55] Yang K.N., Wang H.G., Liu Z.Y. (2021). Evaluation on mechanical properties of high-performance biocomposite bridge deck structure: A review. Polym. Compos..

[56] Reale T., O’Connor A. (2012). A review and comparative analysis of corrosion-induced time to first crack models. Constr. Build. Mater..

[57] Rahman M.M., Islam M.A. (2022). Application of epoxy resins in building materials: Progress and prospects. Polym. Bull..

[58] Wang C., Wang Z., Liu S., Luo H., Fan W., Shi N., Wang H. (2021). Research progress of new carbon nanomaterials used in organic anticorrosive coatings. Surf. Technol..

[59] Moon H.Y., Shin D.G., Choi D.S. (2007). Evaluation of the durability of mortar and concrete applied with inorganic coating material and surface treatment system. Constr. Build. Mater..

[60] Zhang B. (2020). Influencing factors of chloride ion permeability resistance of concrete in cold and arid regions of northwest China. J. High. Trans. Res. Dev..

